# Effects of cueing interventions on online learning outcomes: a meta-analytic review of 40 studies

**DOI:** 10.3389/fpsyg.2026.1717604

**Published:** 2026-03-24

**Authors:** Yan Ma, Xiaoqing Hu, Qirong Peng, Luo Wang

**Affiliations:** Wisdom Education Research Institute, Chongqing Normal University, Chongqing, China

**Keywords:** learning outcomes, meta-analysis, online learning, social cues, visual cues

## Abstract

As a key element in multimedia learning design, cues influence learning outcomes through psychological mechanisms such as attention guidance, cognitive load regulation, and learning motivation stimulation. To systematically examine the effects of different cue interventions on online learning outcomes, this meta-analysis integrated 40 experimental and quasi-experimental studies (*N* = 5,049) published between 2000 and 2025. It conducted in-depth analyses focusing on moderating variables such as educational level, learning material type, and cue type. Cue types encompassed social cues (teaching proxies, teaching gestures), visual cues (subtitles, color, bold, highlighting), and combined cues (social and visual cues). Findings indicate: (1) Cue interventions significantly enhanced online learning outcomes at a moderate effect size (*p* < 0.001); (2) Social cues (g = 0.634) significantly outperformed visual cues (g = 0.261) in enhancing effects, while combined cues exhibited a negative effect (g = −0.635); (3) Learning outcomes were significantly moderated by variables including educational level, learning materials, assessment tools, total gaze duration, and instructional video length. Collectively, these findings elucidate the influence mechanism of cue intervention, aiming to provide theoretical insights and practical guidance for optimizing online learning environments and enhancing student learning outcomes.

## 1 Introduction

With the deepening development of the global digital economy and the accelerated advancement of educational digital transformation, online learning has become a core force reshaping the educational ecosystem. As a mainstream form of online instruction, instructional videos are widely applied across diverse scenarios such as MOOCs, micro-lectures, and smart classrooms. However, learners often face challenges like distraction, cognitive overload, and insufficient engagement during video-based learning, which have become key obstacles and deep-seated challenges to enhancing online learning quality. To address these issues, researchers increasingly focus on how refined instructional design can guide students’ cognitive processes. Among these approaches, “cues” have gained significant attention as a critical intervention strategy. Cues refer to instructional tactics that consciously direct learners’ attention to key information through social or visual prompts within multimedia learning environments, thereby promoting cognitive integration and knowledge construction (De Koning et al., 2007). Psychologically, cues demonstrate significance in three primary ways: First, they capture learners’ attention, thereby simplifying visual search during cognitive processing (Sun and Nembhard, 2025). Second, they highlight relevant information while suppressing distractions, regulating cognitive load and alleviating processing pressure from multimodal materials. Third, in applications represented by social cues, teacher gestures—such as symbolic, metaphorical, and indicative gestures—can direct attention, reduce cognitive load, enhance video learning, and thereby strengthen learners’ sense of social presence and learning motivation.

Despite substantial empirical research on cue interventions both domestically and internationally, three limitations persist: First, existing literature predominantly focuses on visual cues, with limited exploration of social cues and their combined effects; Second, considerable research discrepancies persist: while some studies demonstrate significant learning enhancements through cues (Tabbers et al., 2004; Zheng et al., 2022; Huangfu et al., 2024), others indicate their effects are moderated by multiple variables and may even yield negative outcomes (Crooks et al., 2012; Jaeger and Fiorella, 2023). Finally, current research lacks a systematic meta-analysis to synthesize empirical evidence on cue effects in online learning contexts.

Therefore, this study conducts a meta-analysis of 40 experimental and quasi-experimental studies from Chinese and international literature published between 2000 and 2025. It systematically examines the differential effects of visual cues (color, arrows, bold text, subtitles) and social cues (teacher gestures, instructional proxies) on student learning outcomes in online settings. It further explores the roles of moderating variables such as educational level, subject type, learning materials, measurement methods, total gaze duration, and instructional video length. The study aims to reveal: (1) Cues exert differential effects on learning outcomes across varying variable combinations; (2) Cues regulate learners’ intrinsic cognitive processes at the psychological learning mechanism level; (3) Provide theoretical foundations and practical guidance for designing and optimizing online video learning, thereby advancing the scientific development and high-quality growth of digital educational resources.

## 2 Research design

### 2.1 Research methods and tools

In recent years, the application of meta-analysis in the field of education has experienced explosive growth (Fang and Lei, 2025). Meta-analysis is a quantitative research method within systematic reviews that integrates the results of multiple studies within a specific domain to derive more accurate and universally applicable conclusions (Glass, 1976). This study employs CMA3.3 (Comprehensive Meta Analysis 3.3) software as the meta-analysis tool. This software statistically integrates and reanalyzes data from multiple studies, specializing in calculating effect sizes from statistical data and conducting significance analyses based on these calculations. Relevant data from sample articles—such as sample size N, pre- and post-test standard deviation SD, and mean values—were input into CMA3.3 to derive corresponding effect sizes. Given the small sample sizes in intervention studies and variations in experimental designs and measurement tools across research, this study employed the standardized mean difference Hedges’ g as the effect size metric to assess the impact of interventions on online learning outcomes.

### 2.2 Literature search and screening

**Figure 1:**
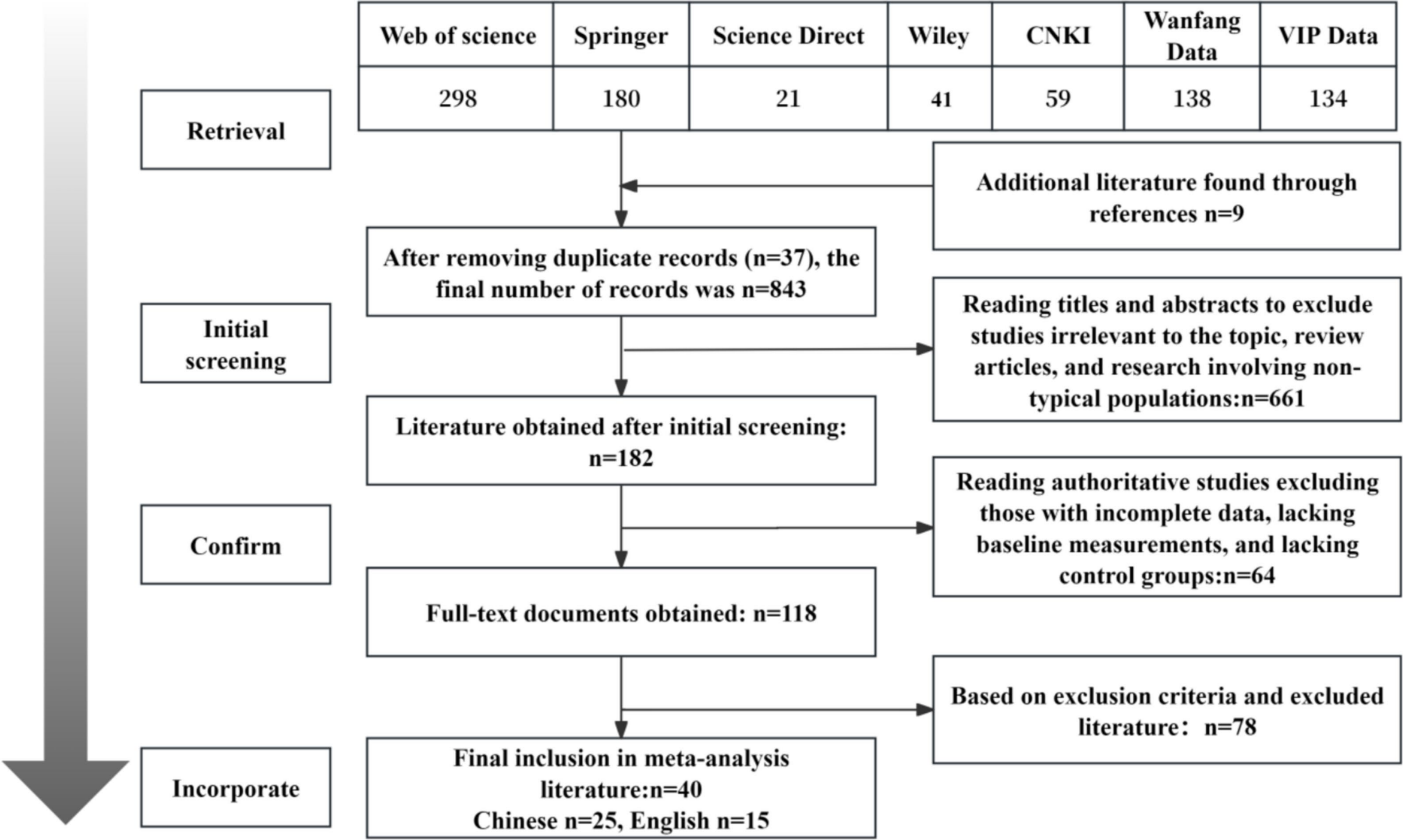
Literature search and screening process diagram.

This study conducted precise searches in Chinese databases such as CNKI, Wanfang, and VIP using keywords including “visual cues,” “social cues,” “cue types,” “subtitles,” “attention guidance,” “videos,” and “online learning,” focusing on the themes of cue types and learning effectiveness. Simultaneously, searches were performed in major English databases including Web of Science, Springer, ScienceDirect, and Wiley. Keywords included “cueing,” “cues,” “attention guidance,” “subtitle,” “caption,” “multimedia learning,” and “online learning,” covering the time span from January 2000 to August 2025. A retrospective literature review method was employed to conduct comprehensive searches based on reference lists. Supplementary searches were also conducted in Google Scholar. The literature screening flowchart is shown in Figure 1.

For the retrieved relevant studies, the following criteria were primarily used to screen them for potential inclusion in the meta-analysis:

(1) The literature must be empirical research on the effects of cues on online learning, with clearly defined sample sizes (no fewer than 10 participants), excluding purely theoretical or review articles.
(2) Detailed pre- and post-test data enabling effect size calculation were reported (mean and standard deviation for both experimental and control groups), excluding data derived from structural equation modeling, regression analysis, or other statistical methods.
(3) Measurement tools assessing learning outcomes were described.
(4) Studies involving typical populations were included, while those targeting special populations were excluded.
(5) For studies with duplicate data publications, only one version was included.

Ultimately, 40 studies meeting the above criteria were identified, yielding 51 distinct effect sizes.

### 2.3 Effect size calculation

This study employs the mean difference in learning outcomes between the experimental and control groups following cue intervention as the effect size. When calculating the effect size, data from pre- and post-intervention measures were recorded. First, the change in scores for each group between pre- and post-intervention was calculated separately. Subsequently, the difference in these changes between the two groups was computed. Given the small sample sizes in intervention studies and variations in experimental designs and assessment tools across different research, this study adopted the standardized mean difference Hedges’ g as the effect size measure. An effect size is considered small when 0.2 ≤ g < 0.5; moderate when 0.5 ≤ g < 0.8; and large when g ≥ 0.8 (Kobayashi, 2024).

### 2.4 Literature coding

Following the screening criteria outlined above, the literature ultimately included in the meta-analysis was coded as follows: (1) Basic literature information (author names, publication dates); (2) Analysis primarily focused on undergraduate and graduate student segments, based on the classification by Wang et al. (2025); (3) Cue interventions were categorized into visual cues and social cues based on Zhao and Hou (2024); visual cues were further subdivided into color contrast, highlighting, arrow markers, and caption annotations following Li (2025), while social cues were classified into teaching proxies and teaching gestures per Fuxing et al. (2025); (4) Following Wei et al. (2024)‘s classification, analysis primarily covers learning materials and subject categories (Wei et al., 2024); (5) Referencing Xie et al. (2016)‘s coding framework, analysis focuses on measurement tools, male ratio, total gaze duration, and video length as moderating variables (Xie et al., 2016).

Coding adheres to the following principles: (1) For each independent sample in the original literature, derive one effect size. If multiple independent samples exist, code them separately; (2) If multiple pre- and post-test means and standard deviations for cue-learning effect measurements are reported, use the mean for processing; (3) For eye-tracking studies, the mean total fixation duration in the region of interest (ROI) recorded by the eye tracker was directly extracted for coding. The ROI was uniformly defined as the key information area marked by the cue; (4) For non-eye-tracking studies where no duration data related to attention fixation was reported, the study was excluded from the meta-regression analysis of total fixation duration but included in tests of other moderator variables. To ensure coding validity, two graduate students independently coded each study, and their results showed no significant discrepancies. When calculating effect sizes for moderators, insufficient sample sizes across groups may compromise results. Therefore, internal conditions within each moderator variable could not be coded individually. Instead, they were grouped based on common characteristics across literature samples to better understand their internal influences (Zhong and He, 2023). Selected literature coding results are presented in Table 1.

**Table 1:** Coding of cue-intervention literature in online learning.

Title of publication (year of publication)	Sample size	Educational stage	Learning materials	Cue type	Subject category	Measuring tools	Effect size (Hedges’g)
Qu (2024)	40	Other	JX	S	W	YD, LB	0.55
Yang et al. (2024)	60	US	JX	S	W	YD, LB	0.93
Chuchu (2022)	128&64	US	JX	C	W	LB	0.02&0.10
Sha (2017)	91	US	JX	C	W	YD、LB	0.61
Hui (2022)	80&86	US	JX	C	W	LB	0.54&0.23
Yang et al. (2019)	360	US+GS	JX	S	G	YD、LB	0.36
Anran et al. (2021)	62	US	JX	C	W	YD	−0.50
Luo (2025)	40	US	JX	C	W	Other	−0.04
Wang (2022)	134	US+GS	JX	S + C	L	YD	−1.48
Pan (2024)	127	US+GS	JX	S	W	YD	0.37
Chen (2023)	48	US	JX	C	L	YD、LB	−0.07
You (2024)	30	US	JX	C	W	Other	0.02
Wang et al. (2024)	114&87	US	ZC	C	L	Other	0.50&1.05
Qiao et al. (2025)	79	US	JX	S	L	Other	0.53
Han (2022)	188	US	JX	C	W	LB	0.1
Zhao (2021)	61&66	US	DC	C	L	YD	0.58&0.67
Li et al. (2024)	92	US+GS	JX	S	W	LB	0.35
Li et al. (2019)	48	Other	Other	S	G	Other	0.28
Wang (2016)	99&99	US	ZC	S	W	Other	3.08&3.23
Chen (2023)	80	US	JX	S	L	YD	0.46
Ma (2023)	79&76	US	DC&JX	S	G	YD	0.89&0.62
Wang et al. (2015)	51	US	DC	C	G	YD	0.02
Aie (2020)	84	US	JX	C	G	YD、LB	−0.51
Lin (2015)	63&64&64	US+GS	DC	S + C	W	YD、LB	−0.28&-1.25&-0.24
Zhao (2024)	159	Other	JX	S	L	LB	−0.12
Heidig et al. (2024)	26&53&38	US	JX	S	W	LB	0.36&0.53&-0.02
Farrell et al. (2024)	130	US+GS	JX	S	W	LB	0.08
Krieglstein et al. (2023)	84	US	DC	S	L	LB	−0.06
Kühl et al. (2025)	126&219	US	JX	S	G	LB	−0.18&0.15
Liew and Zin (2017)	72	US	JX	S	W	LB	0.64
Cintrón-Valentín and García-Amaya (2021)	369	US	JX	C	W	Other	0.12
Meusel et al. (2024)	191	Other	ZC	C	W	LB	0.17
Liu et al. (2021)	110	US	JX	C	L	YD	0.72
Liu et al. (2021)	38	GS	JX	C	G	Other	0.85
Beege et al. (2021)	138	Other	ZC	C	G	Other	1.06
Pannatier and Bétrancourt (2024)	131	US	JX	C	W	LB	0.09
Chai and Erlam (2008)	20	US	JX	C	W	Other	1.45
Lin et al. (2015)	126	US	DC	C	L	Other	0.27
Li et al. (2024)	40	US	JX	C	G	Other	−0.08
Sylvia et al. (2023)	135	US	JX	C	L	YD	−0.66

US, Undergraduate students; GS, Graduate students; JX, Teaching videos, ZC-Graphic materials, DC-Animations, S-Social cues, C-Visual cues, L-Science, G-Engineering, W-Liberal arts, YD-Eye tracker, LB-Scales.

## 3 Analysis of research findings

### 3.1 Heterogeneity test

The results of the heterogeneity test determine the appropriate model for analysis. This study employed the Q test and I^2^ statistic for heterogeneity assessment, with the following criteria: low heterogeneity (0% < I^2^ < 50%), moderate heterogeneity (50% ≤ I^2^ < 75%), and high heterogeneity (I^2^ ≥ 75%) (Xu et al., 2024). Heterogeneity testing was performed using CMA 3.3 software, with results presented in Table 2. The Q-value was 431.542 (*p* < 0.01), and the I^2^ value was 88.41%, indicating significant heterogeneity among the studies. Borenstein et al., (2021) recommended selecting a random-effects model when I^2^ > 75% (Wolfe et al., 2016) and a fixed-effects model when 0 ≤ I^2^ ≤ 75%. Therefore, the random-effects model should be employed for meta-analysis when calculating effect sizes in this study.

**Table 2:** Results of heterogeneity tests.

Model	K	N	Heterogeneity				Tau-squared			
			Q_B_	df	*P*	I-squared	Tau-squared	SE	Variance	Tau
Random effects model	40	5,049	431.542	50	0.000	88.414	0.373	0.099	0.010	0.611

K, represents the number of independent effect sizes; N, denotes the sample size; QB, represents the heterogeneity test statistic.

### 3.2 Publication bias test

**Figure 2:**
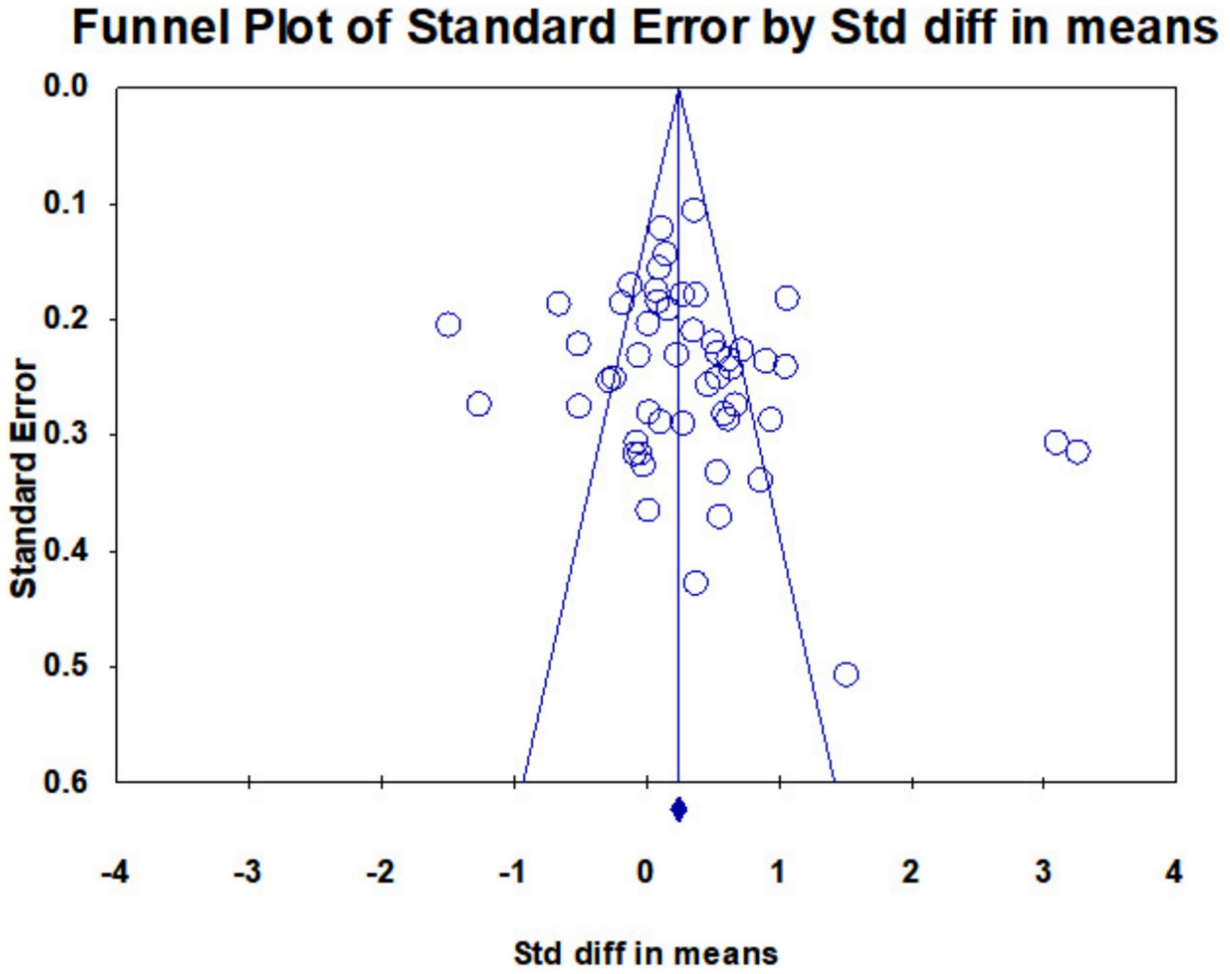
Funnel diagram of the impact of cue intervention on online learning outcomes.

The publication bias test primarily examines whether publication bias exists in the sample. First, an effect size distribution funnel plot test was conducted, with results shown in Figure 2. The studies were predominantly concentrated in the upper portion of the funnel plot, with fewer studies located in the lower section. Research was evenly distributed across both sides of the funnel plot, exhibiting a largely symmetrical distribution. This indicates a low likelihood of publication bias in the meta-analysis. Therefore, the Classic Fail-safe N method was employed to assess publication bias, quantitatively estimating the publication bias level at *p* = 0.05. Following Rothstein et al., a threshold of 5 K + 10 (where K represents the number of studies) was used for judgment. For this study, K = 40. Thus, a Fail-safe N exceeding 5*40 + 10 = 210 indicates no significant publication bias. Results show a Fail-safe N of 971, substantially above the threshold, confirming no publication bias in this meta-analysis. Furthermore, Egger’s regression coefficient analysis revealed an intercept of 1.95 (*p* > 0.05), with the *p*-value being non-significant. This confirms the absence of publication bias.

### 3.3 Main effects analysis

This meta-analysis included 40 studies and 51 independent effect sizes, involving a total of 5,049 participants. Using CMA 3.3 to conduct a random-effects model analysis, the results showed that the effect size of cue-based interventions on online learning outcomes was 0.320 (95% CI [0.138, 0.502], *p* < 0.001), indicating that cue-based interventions enhance online learning outcomes. Detailed results are presented in Table 3.

**Table 3:** Results of main effect tests.

Model	K	N	Point-estimate	95%CI	*P*	Z-value
Random effects model	40	5,049	0.320	[0.138–0.502]	0.000	3.450

### 3.4 Moderation effect analysis

To further explore the differential effects of cue interventions on students’ online learning outcomes, this study conducted subgroup analyses using educational level, learning materials, cue type, subject category, and measurement tools as moderating variables, with results presented in Table 4 below. Significant between-group effects were observed for educational level, learning materials, cue type, and measurement tools (*p* < 0.05), indicating these factors significantly moderate the effectiveness of cue interventions. In contrast, the between-group effect for subject category was non-significant (*p* > 0.05), suggesting the cue mechanism exhibits strong stability across disciplinary contexts. Additionally, this study examined continuous moderators including total gaze duration, gender ratio, and teacher video length. The regression coefficient for total gaze duration on effect size was significant (p < 0.05), while the regression coefficient for gender ratio was not significant (*p* > 0.05). The regression coefficient for instructional video duration was significant (*p* < 0.05), consistent with cognitive load theory predictions. Videos that are too short may fail to allow learners to fully integrate information in working memory, while excessively long videos may lead to attention dispersion and cognitive overload, thereby diminishing cue effectiveness.

**Table 4:** Results of moderator effect analysis.

Subgroup variable		K	Hedges’g	95%CI	Qb(df)	*P*
Educational stage	Undergraduate	38	0.419	[0.211, 0.628]	6.558 (2)	0.038
	Undergraduate students+ Graduate Students	8	−0.247	[−0.734, 0.240]		
	Others	5	0.519	[−0.021, 1.060]		
Study materials	Instructional video	35	0.174	[0.014, 0.333]	8.424 (3)	0.038
	Illustrated materials	6	1.502	[0.592, 2.413]		
	Animation materials	9	0.073	[−0.327, 0.473]		
	Others	1	0.280	[−0.289, 0.849]		
Cue type	Visual cues	26	0.261	[0.078, 0.444]	12.427(2)	0.002
	Social cues	20	0.634	[0.320, 0.949]		
	Combination cue	5	−0.635	[−1.286, 0.016]		
Subject category	Engineering	11	0.315	[0.034, 0.595]	9.427(3)	0.024
	Science	13	0.179	[−0.205, 0.563]		
	Liberal Arts	27	0.399	[0.117, 0.681]		
Measuring tools	Eye tracker category	11	0.151	[−0.321, 0.623]		
	Scale-type	17	0.131	[0.029, 0.232]		
	Combination Category	9	0.004	[−0.408, 0.415]		
	Others	14	0.875	[0.396, 1.353]		

#### 3.4.1 Educational level

Participants were grouped based on their educational level across independent studies: undergraduates, mixed groups of undergraduates and graduate students, and others. Subgroup analysis revealed that educational level significantly moderated the effect of cueing interventions on online learning outcomes (p < 0.05). For undergraduates, the effect size of cue intervention on online learning outcomes was g = 0.419, indicating greater effectiveness of cue intervention in online learning for this group (Han et al., 2024). The mixed group of undergraduates and graduate students exhibited a negative effect size of g = −0.247. This may stem from graduate students’ higher cognitive levels and reduced reliance on cues. Additionally, prior research suggests that providing detailed external guidance to knowledgeable learners may hinder their learning and performance. It should be noted that the number of studies included in the graduate subgroup was limited. Therefore, the findings for graduate students should be interpreted as exploratory trends rather than definitive conclusions, and conclusions drawn from this subgroup should be treated with extreme caution.

#### 3.4.2 Cue types

This study categorizes cue types into visual cues, social cues, and combined cues. Among visual cues, subtitles, color, bold text, and highlighting yielded g = 0.289 (see Table 5), indicating a moderate effect. This suggests that while visual cues like color and arrows can rapidly direct attention, their impact remains limited to information selection and offers limited promotion for deep cognitive processing. Therefore, it may be necessary to visually convey verbal instructions on-screen to facilitate the information selection process (Jamet and Fernandez, 2016). Among social cues, instructional proxies (g = 0.610) demonstrated a moderately strong to strong facilitation effect. This indicates that social cues like teacher proxies can simulate authentic teaching scenarios, enhance learners’ emotional engagement and motivation, while complementing verbal explanations to promote knowledge understanding and integration (Li, 2024). However, the combined cue (social cue + visual cue) yielded an effect size g = −0.635, indicating a negative effect. This may stem from cognitive overload due to cue redundancy, where learners must process multiple cues simultaneously, potentially disrupting normal cognitive processing. Given the small sample size (k = 8), caution is warranted regarding this negative effect.

**Table 5:** Specific cue moderation effect tests.

Subgroup variable			K	Hedges’g	95%CI	Qb(df)	*P*
Cue type	Social cues	Teaching agent	7	0.610	[−0.342, 1.561]	8.334 (3)	0.040
		Teaching gestures	12	0.294	[0.091, 0.496]		
	Visual cues	Subtitles, color, Bold, highlight	31	0.289	[0.066, 0.512]		

#### 3.4.3 Learning materials

This study categorized the learning materials used in the experiment into instructional videos, text-and-image materials, animations, and others. Learning materials significantly moderated the effects of online learning cues. Among these, text-and-image materials yielded an effect size of g = 1.502. These materials featured low information density and minimal visual complexity, allowing cues to clearly highlight key content and guide learners toward efficient information processing. The effect size for instructional videos was g = 0.174. Videos incorporate multimodal information such as dynamic visuals and audio, making learners prone to distraction. The effect size for animated materials was g = 0.073, showing no significant effect. Although text-and-image materials and animated materials showed divergent trends in small-effect paths, given the small sample sizes and uneven distribution across the two subgroups, this study refrained from pairwise comparisons of effect sizes. Only descriptive statistics were reported to avoid overgeneralization due to sample bias.

#### 3.4.4 Measurement tools

Based on the literature review, most studies employ eye-tracking devices to objectively evaluate the learning outcomes of online learners, while a smaller number report student learning effectiveness through self-assessment questionnaires or scales. Measurement tools serve as the core vehicle for assessing whether instructional cues effectively guide learning. This study categorizes measurement tools into four main types based on the degree of objectivity in measurement attributes and the form of tool usage: eye-tracking devices, scales, combined measurement approaches, and other methods. This classification demonstrates empirical applicability and theoretical soundness. Theoretically, it follows the cognitive assessment logic of “objective measurement, subjective measurement, and combined objective-subjective measurement” (van et al., 2009), aligning with the components of online learning effectiveness: attentional behavior, subjective perception, and overall outcomes. Empirically, the measurement tools included in this study can be clearly categorized into four distinct usage forms: single objective tools, single subjective tools, combined objective-subjective tools, and others, with no ambiguity. This ensures the statistical validity of moderation effect tests. The study incorporated seven mainstream measurement tools (see Table 6). Through effect size differences, it revealed a strong correlation between tool type and cue assessment efficacy. Eye trackers, by recording metrics such as “gaze duration” and “gaze trajectory,” directly reflect the effectiveness of cues in guiding attention, making them the most effective measurement tool category in the study: Eyelink eye tracker effect size g = 0.347, representing a small to medium positive effect. This tool is widely used to capture learners’ immediate attentional responses to cues, such as tracking gaze focus guided by “teacher gestures” (social cues) or “color highlighting” (visual cues). Tobi iX120 Eye Tracker: Effect size g = 0.352. The effect size for eye tracker measurement tools (g = 0.347) is significantly higher than that for questionnaire-based tools (g = 0.105). This classification exhibits distinct mechanistic characteristics in influencing learning effectiveness research outcomes. Eye tracker tools focus on the objective behavioral dimension of attention allocation, precisely detecting how cues guide learners’ visual attention. Thus, their effect sizes more accurately reflect the essence of cue-driven attention guidance. Scale-based tools concentrate on subjective dimensions of learning experience and cognitive load, making them susceptible to learner metacognitive biases. Consequently, their effect sizes tend to be lower and more prone to fluctuation. Combined measurement approaches attempt to integrate subjective and objective dimensions, but due to differences in assessment logic between eye-tracking’s objective behavioral data and scales’ subjective perception data, the merged data fails to produce synergistic effects. Other tools primarily utilize non-standardized tests, assignments, and similar instruments, concentrating on the comprehensive dimension of knowledge mastery. These tools align closely with the “key information guidance” objective of cue interventions, resulting in the largest effect sizes.

**Table 6:** Test of measurement tool adjustment effects.

Subgroup variable			K	Hedges’g	95%CI	Qb(df)	*P*
Measuring tools	Eye tracker category	Eyelink Eye Tracker	4	0.347	[0.186, 0.509]	14.699 (6)	0.023
		SMI RED 250 Eye Tracker	4	−0.192	[−1.227, 0.893]		
		Tobi iX120 Eye Tracker	6	0.352	[−0.198, 0.902]		
	Scale-type	7-point Likert scale	12	0.105	[−0.060, 0.269]		
		Video Course Learning Satisfaction Scale (PAAS) Questionnaire	6	0.103	[−0.064, 0.270]		
	Combination measurement category	Eyelink Eye Tracker, PAAS Questionnaire	5	−0.071	[−0.801, 0.660]		
	Other categories	Others	14	0.875	[0.396, 1.353]		

#### 3.4.5 Gaze duration and instructional video length

To examine the effect of continuous variables on effect size, this study incorporated “total gaze duration” (unit: milliseconds), “instructional video length” (unit: minutes), and “gender ratio” (male proportion) into the meta-regression model. Results indicate: The regression coefficient for total gaze duration is significant (*p* < 0.05), suggesting learners achieve better learning outcomes with longer dwell times in cue zones. The main effect of instructional video duration is significant (*p* < 0.05). Furthermore, existing research indicates that enhanced interactive features may be more effective in longer videos than in shorter ones (Yang et al., 2024). Gender ratio did not significantly predict learning outcomes (*p* > 0.05). However, under high-interaction conditions, female learners exhibited a significantly greater increase in gaze duration than males. Future adjustments to cue density based on gender differences could further optimize the online learning experience.

## 4 Research findings and recommendations

### 4.1 Research findings

Through a meta-analysis of 40 experimental and quasi-experimental studies conducted domestically and internationally between 2000 and 2025, combined with Wei et al. (2024) research on cue facilitation in digital learning, this study systematically examined the effects of cue interventions on online learning outcomes and their moderating mechanisms. The following research conclusions were drawn:

#### 4.1.1 Cognitive load modulation mechanism of learning outcomes

The overall effect test revealed that the comprehensive effect size of cue intervention on online learning outcomes was g = 0.320 (*p* < 0.001), indicating a moderate positive effect. This result demonstrates that cues, as a pedagogical intervention, significantly enhance online learning outcomes, with this facilitative effect exhibiting statistically stable reliability. From a cognitive processing perspective, learning materials containing multiple information elements impose heavy cognitive load (Sweller and Chandler, 1994). Cues guide learners to focus attention on key information, reducing interference from irrelevant details and lowering external cognitive load. This creates conditions for efficient cognitive processing, enabling learners to grasp core concepts and clarify contextual relationships more rapidly. This conclusion also corroborates the eye-tracking findings of Boucheix and Lowe (2010), demonstrating that visual cues more effectively direct attention toward topic-relevant aspects compared to no cues, thereby improving scores on knowledge comprehension. This underscores the importance of cues in cognitive processes (Lowe and Boucheix, 2011). This finding also implies that the value of cues extends beyond short-term learning gains to include their influence on learners’ cognitive strategies.

#### 4.1.2 Mechanism of social cues activating affective motivation

The overall effect size for social cues (teaching proxies, teacher gestures, etc.) reached g = 0.634, representing a medium-to-large effect that significantly outperformed visual cues. Further analysis revealed that the effect size for teaching proxies was g = 0.610, while that for teacher gestures was g = 0.294. This indicates that social cues not only effectively guide attention allocation by simulating interactive behaviors in real teaching scenarios but also foster a sense of interaction and belonging. This enhances learners’ emotional engagement and sustained participation, aligning with the core tenets of social presence theory—that students’ connectedness and learning motivation can be increased through real or imagined interactions with others (Li et al., 2021). In contrast, the combined effect size for visual cues (subtitles, color, highlighting, bold text) was only g = 0.289, while the combination of cues produced a negative effect, confirming the “redundancy effect” proposed by cognitive load theory. When multiple cues are presented simultaneously, learners expend additional cognitive resources processing the relationships between cues, which instead interferes with the processing of core learning content (Scheiter and Eitel, 2015).

#### 4.1.3 Cognitive development mechanisms in educational stages and material differences

The type of learning material significantly influences the effectiveness of cue interventions. Results indicate that graphic-text materials exhibit the strongest cue effect, while animations show the weakest. This suggests that in relatively concise multimedia courseware or graphic-text materials, external cues can more effectively guide learners in allocating limited cognitive resources, thereby enhancing knowledge processing and learning outcomes. However, in animated materials incorporating dynamic images and audio, learners must process multimodal information themselves, diminishing the impact of additional cues and potentially triggering cognitive conflict. Research indicates that in animated contexts, static cues often fail to capture attention as effectively as dynamic cues. Thus, exploring dynamic attention-guiding mechanisms in animations may yield superior learning outcomes (Shen and Lowe, 2009).

Regarding educational level differences, the effect size of cues for undergraduate students (g = 0.419) was significantly higher than that for mixed groups of undergraduates and graduate students (g = −0.247). This discrepancy likely stems from differing cognitive development levels: graduate students possess stronger autonomous information filtering and deep processing abilities, reducing their reliance on external cues and potentially viewing them as redundant information. Undergraduates, however, remain in the developmental stage of cognitive strategies and knowledge construction, requiring external cues to assist in building knowledge frameworks. This finding aligns with Yang et al. (2019), who concluded that teacher gesture guidance enhances video learning effectiveness, particularly for less experienced learners or those with developing cognitive abilities (Heidig et al., 2024).

#### 4.1.4 Mechanisms of effect differences between measurement methods and disciplinary factors

The moderating effect of measurement tools was significant (*p* > 0.05), with eye-tracking instruments demonstrating significantly higher assessment efficacy than questionnaire-based tools: The effect size for the Eyelink eye tracker (g = 0.347) and the Tobi iX120 eye tracker (g = 0.352) was significantly higher than that for the 7-point Likert scale (g = 0.105) and the PAAS cognitive load questionnaire (g = 0.105). Tobi iX120 eye tracker effect size (g = 0.352) were both significantly higher than the 7-point Likert scale (g = 0.105) and PAAS cognitive load questionnaire (g = −0.071). The significantly higher assessment efficacy of eye-tracking tools compared to questionnaire-based tools stems from the “degree of objectivity” of the measurement instruments (van et al., 2009): Eye trackers directly reflect the effectiveness of cues in guiding attention by recording objective metrics such as gaze trajectories and interest area fixation rates, unaffected by learners’ subjective cognitive biases. In contrast, questionnaire-based tools rely on learners’ subjective reports, making them susceptible to “social desirability bias” (e.g., overestimating learning outcomes) or “metacognitive judgment errors” (Bartura et al., 2023). This indicates that eye-tracking technology is more suitable as the core assessment method for cue effects. The cueing effect size for humanities learning (g = 0.399) exceeded that for engineering (g = 0.315) and science (g = 0.179). Humanities knowledge relies more heavily on “key information extraction,” where cues play a more prominent guiding role. Conversely, science and engineering knowledge depend more on “logical deduction and practical application,” where cues assist in locating information but cannot directly facilitate logical processing. This aligns with relevant research findings.

### 4.2 Research recommendations

#### 4.2.1 Control the number of video cues to achieve efficient guidance

The findings on the efficacy differences across various cue types indicate that more cues are not necessarily better. Excessive or poorly designed cues may trigger a “redundancy effect,” increasing learners’ cognitive load and thereby distracting attention and undermining learning outcomes. Therefore, it is essential to rationally control the quantity and variety of cues. In teaching practice, priority should be given to efficient cue presentation methods that significantly enhance attention allocation efficiency and cognitive processing quality, while avoiding ineffective or redundant prompts. Specific implementation can focus on the following three aspects: (1) Dynamic Presentation: In videos such as MOOCs and micro-lectures, present relevant cues in phases according to the learning rhythm. Incorporate social cues like instructor gestures or teaching agent interactions. Instructor social cues may activate learners’ social responses and emotional engagement, thereby enhancing learning motivation. This demonstrates that social cues serve not only as attention-guiding mechanisms but also as crucial psychological mechanisms for fostering learners’ emotional involvement and motivational stimulation. (2) Combination Optimization: Prioritize static visual cues like color highlighting and arrow annotations for text-based materials. Teaching videos should emphasize social cues supplemented by minimal dynamic visual cues. Animations can utilize “camera focus” functions to replace external cues and minimize distractions. (3) Discipline Alignment: Humanities courses may use color coding to distinguish textual hierarchical levels (e.g., red for titles, blue for arguments), complemented by instructor gestures emphasizing key points to help learners organize knowledge frameworks. Engineering courses may employ visual cues like arrow annotations and bold text to illustrate structural relationships and operational procedures. Science courses may integrate instructor gestures guiding formula derivation steps to reinforce logical chain comprehension.

#### 4.2.2 Develop personalized plans in accordance with students’ cognitive patterns

Based on the moderating effect of cue effectiveness across educational stages, differentiated guidance plans should be designed for learners at different levels. Undergraduate students are in a critical phase of cognitive and learning strategy development, lacking mature information filtering and knowledge integration skills. Therefore, moderate-intensity cue interventions should be designed, with explicit cue markers placed at knowledge challenges to aid in constructing conceptual frameworks. Graduate students possess strong independent information filtering abilities and rely less on external guidance. Concise visual cues can be provided at challenging content points, such as interdisciplinary theoretical integration and complex conceptual connections. From the perspective of cue designers, video developers should align cues closely with learning objectives and knowledge points during scriptwriting and production stages, tailored to learners at different educational levels. Through collaborative design involving developers, educators, and teams, cue strategies can be better matched to learners’ cognitive development stages, achieving genuine personalized support.

#### 4.2.3 Dynamically adjust intensity based on learning behavior data

As online learning platforms accumulate vast amounts of learning process data—such as study duration, response performance, and click paths—machine learning algorithms can be leveraged to build predictive models for cue effectiveness. This enables personalized prediction and intervention for learners. Simultaneously, eye-tracking technology can optimize cue design. During course development, eye trackers monitor metrics like gaze duration, fixation paths, and first-gaze latency on guidance content. This process eliminates ineffective cues and selects guidance methods aligned with learners’ cognitive rhythms. For different learners, analyzing average fixation duration on guidance content allows adjusting cue presentation duration and visual intensity. This ensures guidance effectively captures attention without overloading cognitive resources. Future developments may even include intelligent cue recommendation systems: these systems would dynamically detect learners’ attention states and progress, pushing the most suitable cue types and quantities in real time. This would deliver truly personalized, precise, and adaptive learning support.

#### 4.2.4 Integrating multimodal brain-computer technologies to uncover psychological and neural mechanisms

Combining brain imaging techniques (such as EEG, fNIRS, fMRI) to detect the effects of cueing interventions on neural activity, thereby revealing the brain mechanisms underlying attention regulation, cognitive load modulation, and learning motivation activation. For instance, functional magnetic resonance imaging (fMRI) possesses high spatial resolution, enabling the mapping of activation patterns in deep brain regions and aiding in uncovering the mechanisms by which cues influence memory and retrieval effects. Meanwhile, functional near-infrared spectroscopy (fNIRS) measures changes in cerebral oxygenation levels within cortical regions. When cues are redundantly designed or cognitive overload occurs, fNIRS can monitor decreases in prefrontal cortex oxygenation, providing neurobiological evidence. In scenarios requiring subjective evaluations, standardized scales with validated reliability and validity (e.g., the PAAS cognitive load questionnaire) should be prioritized to avoid issues of non-comparability and insufficient reliability associated with self-developed scales. By integrating subjective and objective measures through a multi-level approach, we can not only effectively mitigate interference from social desirability effects and metacognitive biases but also provide more scientific and generalizable empirical evidence for designing and optimizing cue-based interventions.

## 5 Research outlook

### 5.1 Research limitations

Limitations of this study: (1) Cue intervention types are diverse and structurally complex, with varying classifications of their components across studies. This research combined visual, social, and combined cues to examine their overall effects, failing to distinguish the unique impact of different components on learning outcomes; (2) Subgroup analyses exhibited uneven sample distribution, with undergraduate studies vastly outnumbering graduate and other educational levels, potentially obscuring stage-specific differences; (3) Measurement tools were heterogeneous, with some studies employing self-developed or adapted scales for learning outcomes, cognitive compliance, and learning satisfaction. These were categorized as “other tools” during coding, limiting identification of optimal measurement approaches; (4) Non-eye-tracking studies equated total gaze duration with “cue-relevant dwell time.” This indirect metric fails to accurately reflect learners’ actual gaze trajectories and attentional focus. Furthermore, some non-eye-tracking studies omitted relevant duration data, resulting in sample loss that may compromise the precision of analyses examining the moderating effect of total gaze duration. Future work should standardize tools to deepen understanding of the underlying mechanisms linking cue interventions to learning outcomes.

### 5.2 Research prospects

Future studies may further expand sample sizes and integrate multimodal data (e.g., EEG, fNIRS) to deeply elucidate the cognitive neural mechanisms underlying cues. Longitudinal research should also be conducted to examine the long-term learning effects of cues. By integrating multi-modal tools like eye-tracking, EEG, and fNIRS, researchers can analyze cue mechanisms across three dimensions—“attention, cognition, and emotion.” For instance, fNIRS measurements of blood oxygen saturation changes could reveal the extent of decreased blood oxygenation in the dorsolateral prefrontal cortex during cognitive overload induced by combined cues, providing neuroscientific evidence for cue redundancy. Furthermore, future research may integrate large-scale behavioral data from online learning platforms (e.g., study duration, click trajectories, test scores) with machine learning and predictive modeling methods to establish a multimodal data-driven predictive framework for cue effects. This cross-level, cross-methodological research approach will not only deepen our understanding of the psychological and neural mechanisms of cues but also provide empirical support for personalized instructional resource design and intelligent learning environment optimization.
